# Investigating the Relationship Between Personality Traits and Treatment Adherence With the Mediating Role of Coping Styles in Adults With Presbycusis: A Cross‐Sectional Study

**DOI:** 10.1002/hsr2.72955

**Published:** 2026-08-02

**Authors:** Zahra Davari, Sara Zamiran, Leila Ganji, Malek Amini, Shirin Keyvan Nasab, Marzieh Pashmdarfard

**Affiliations:** ^1^ Department of Educational Psychology and Rehabilitation Counselling, Tehran Medical Branch Islamic Azad University Tehran Iran; ^2^ Department of Occupational Therapy, School of Rehabilitation Shahid Beheshti University of Medical Sciences Tehran Iran; ^3^ Department of Occupational Therapy, Rehabilitation Research Centre and School of Rehabilitation Sciences Iran University of Medical Sciences Tehran Iran; ^4^ Department of Occupational Therapy, School of Rehabilitation, Imam Hossein Hospital Shahid Beheshti University of Medical Sciences Tehran Iran

**Keywords:** coping styles, hearing impairment, hearing loss, personality traits, presbycusis, quality of life, treatment adherence

## Abstract

**Background:**

Presbycusis, an age‐related hearing disorder, affects communication, psychosocial functioning, and quality of life. Because its management requires long‐term engagement with rehabilitation and medication routines, psychological factors such as personality traits and coping styles may influence treatment adherence. This study investigated the relationship between personality traits and adherence in adults with presbycusis, with coping styles examined as a mediating variable.

**Methods:**

A descriptive cross‐sectional study was conducted among adults diagnosed with presbycusis recruited from hospitals and audiology centers across Tehran, Iran (November 2024–March 2025). Participants completed the Personality Insights Questionnaire, the Coping Styles Questionnaire, and the Morisky Medication Adherence Scale (MMAS‐8). Partial Least Squares Structural Equation Modeling (PLS‐SEM) was used to assess direct and indirect effects and evaluate model reliability, validity, and predictive accuracy.

**Results:**

A total of 384 adults participated. Personality traits showed a significant positive association with coping styles (*β* = 0.652, *p* < 0.05), and coping styles significantly predicted treatment adherence (*β* = 0.379, *p* < 0.05). The indirect effect of personality traits on adherence through coping styles was significant (*β* = 0.247; *t* = 6.878). Model indices demonstrated acceptable reliability, convergent and discriminant validity, and moderate predictive relevance.

**Conclusion:**

Personality traits influence treatment adherence in adults with presbycusis both directly and through coping styles. Incorporating personality‐based and coping‐focused strategies into hearing rehabilitation may strengthen adherence and improve long‐term outcomes.

## 1 Introduction

Hearing impairment is one of the most common global health conditions [1], affecting an estimated 450 million people worldwide and contributing substantially to communication difficulties, psychosocial distress, and reduced quality of life [2, 3, 4]. Presbycusis, the age‐related form of sensorineural hearing loss, is highly prevalent among older adults and is characterized by reduced hearing sensitivity, impaired speech perception in noise, slowed auditory processing, and difficulty with sound localization [5, 6, 7]. Its consequences extend beyond communication, contributing to social isolation, depression, cognitive decline, falls, and increased mortality [8, 9]. Although approximately two‐thirds of adults aged 70 years and older experience presbycusis, only a minority receive appropriate therapeutic intervention, despite the chronic and progressive nature of the condition and the need for long‐term adherence to rehabilitation and medication routines [9, 10, 11].

Given the chronic and progressive nature of presbycusis, effective long‐term management is essential. Treatment adherence is essential for optimizing outcomes in presbycusis, yet adherence rates remain suboptimal [12]. Psychological factors—including personality traits, emotional regulation, disease acceptance, and coping strategies—play a central role in shaping health behaviors and may influence whether individuals consistently follow treatment recommendations [13, 14]. Personality traits are stable psychological characteristics that affect decision‐making, interpersonal functioning, and health‐related behaviors [15, 16]. Traits such as conscientiousness, extraversion, agreeableness, and neuroticism have been linked to adherence patterns in chronic disease populations, with conscientiousness associated with proactive health behaviors and neuroticism linked to stress‐related avoidance [13, 17]. While personality traits are partly innate and relatively stable over time, research indicates that they can change due to life experiences and environmental factors and may also significantly affect the selection and application of specific coping styles when facing chronic health challenges [18, 19].

Coping styles, defined as the cognitive and behavioral strategies individuals use to manage stress and daily challenges, may serve as a mechanism through which personality traits influence adherence [20, 21]. Problem‐focused coping is generally associated with adaptive health behaviors, whereas emotion‐focused or avoidant coping may undermine adherence [22, 23]. Prior research suggests that personality traits shape coping tendencies, and coping styles in turn influence adherence across chronic conditions [24, 25]. However, limited evidence exists regarding these relationships in adults with presbycusis, despite the long‐term nature of hearing rehabilitation and the psychological demands associated with communication difficulties.

Understanding the relationship between personality traits, coping styles, and treatment adherence is essential for developing effective treatment programs for adults with presbycusis [21]. Despite the importance of these psychological factors, limited research has examined their interrelationships in adults with presbycusis. Given these gaps, this study examined whether personality traits predict treatment adherence in adults with presbycusis and whether coping styles mediate this relationship. We hypothesized that personality traits would exert both direct and indirect effects on adherence through coping styles. Understanding these pathways may support the development of psychologically informed rehabilitation strategies that enhance adherence and improve outcomes for individuals with presbycusis.

## 2 Materials and Methods

### 2.1 Study Design

This descriptive cross‐sectional study examined the relationship between personality traits and treatment adherence, with coping styles evaluated as a mediating variable. Reporting followed the STROBE checklist [26].

### 2.2 Setting

Data were collected between November 2024 and March 2025 from adults with presbycusis referred to hospitals and audiology centers across Tehran, Iran. Recruitment occurred at Imam Khomeini Hospital (west), Imam Hossein Hospital (east), Amir Aalam Hospital (central), and Loghman Hospital (south). After obtaining institutional permission, questionnaires were distributed through head nurses using Google Forms, and participants also received online versions to facilitate completion.

### 2.3 Participants

Eligible participants were adults aged ≥ 18 years diagnosed with presbycusis by an ENT specialist. Inclusion criteria required bilateral and symmetrical sensorineural hearing loss and no history of head injury or ear surgery. Exclusion criteria included outer or middle ear disease. All participants received an explanation of the study purpose, had opportunities to ask questions, and provided written informed consent.

### 2.4 Variables

In this study, personal characteristics of participants were collected using a demographic questionnaire. Personality traits and coping styles were measured through the Personality Insights and Coping Styles Questionnaire. Treatment adherence was evaluated through a medication adherence measure.

### 2.5 Measures

We used the following measures to collect data:

1. *Demographic Questionnaire*: In the researcher‐made demographic questionnaire, the demographic information of the participants, including age, gender, education level, smoking status, name of hospital, and history of illness was collected.
2. *Personality Insights*: This questionnaire was developed by Bass, Valenzi, and Eldridge (1975) and includes 27 questions in 4 components, which provide an insight into how individuals perceive themselves in relation to workplace behaviors, decision‐making, and interpersonal dynamics. It includes a series of statements rated by respondents to reflect their agreement or self‐assessment, forming a profile of personality and leadership tendencies, for example, “I consider myself someone who plans tasks carefully,” which reflects the style of items used to assess personality traits. The scoring method was based on a 5‐point Likert scale (completely agree to completely disagree) [27], and its reliability was reported based on Cronbach's alpha of 0.74. Its face and content validity were also confirmed by experts [28].
3. *Morisky Medication Adherence Measure (MMAS‐8):* This questionnaire, designed by Morisky, Ang and Wood (2008), includes eight questions that assess the level of patients' adherence to medication instructions based on a Likert scale and includes questions such as, “In the past two weeks, has there been a day when you did not take your medications for any reason other than forgetting?”. The MMAS‑8 produces a total score ranging from 0 to 8, where lower scores represent higher adherence and higher scores represent lower adherence. In this scoring structure, most items are dichotomous, with “yes” responses indicating nonadherence and contributing to a higher total score. The mean adherence score in our sample was 0.48, which corresponds to a generally high level of adherence among participants. These studies confirm that the MMAS‐8 continues to be an appropriate and scientifically acceptable measure of medication adherence [29, 30, 31]. The face and content validity of this tool has been reported by expert professors in Iran, and its reliability using Cronbach's alpha has been reported to be 0.68 [32].
4. *Coping Styles Questionnaire*: This questionnaire, developed by Liu (2022), includes 20 items across two dimensions of positive and negative coping styles. It features a seven‐factor structure, with each factor representing a unique coping style, including Withdrawal, Positive Adjustment, Problem‐Solving, Seeking Support, Distraction, Self‐Control, and Negative Coping. Its scoring scale is based on a 5‐point Likert scale ranging from “very little” to “very much” [33].

### 2.6 Study Size

Using Cochran's formula, a minimum sample size of 384 was required. A total of 508 questionnaires were distributed, and 384 valid responses were retained. During data screening (December 2024–January 2025), 124 questionnaires were excluded due to missing data, invariant response patterns, or irrelevant answers, following established guidelines for identifying careless responses [34, 35]. Specifically, 60 questionnaires were excluded due to missing answers exceeding acceptable thresholds, 46 were removed because of consistent response patterns indicating lack of engagement, and 18 were excluded due to irrelevant or contradictory responses. This rigorous screening process reflects our commitment to ensuring high‐quality and reliable data.

### 2.7 Statistical Methods

Data were analyzed using SPSS 23. Descriptive statistics (mean, standard deviation, range) summarized participant characteristics and main variables. Structural relationships among personality traits, coping styles, and treatment adherence were examined using partial least squares structural equation modeling (PLS‐SEM). Model evaluation included the following: indicator reliability (outer loadings), internal consistency (composite reliability), average variance extracted (AVE), discriminant validity (Fornell–Larcker criterion), variance infaltion factor (VIF), predictive accuracy (*R* ^2^), predictive relevance (*Q* ^2^), and standardized root mean squared residual (SRMR). PLS‐SEM was selected due to its robustness to nonnormal data and suitability for mediation analysis.

### 2.8 Ethical Considerations

This study is approved by the ethics committee of the Islamic Azad University of Tehran, Iran (IR.IAU.TMU.REC.1403.402). The patient records and information were anonymized and de‐identified before analysis. All participants provided written informed consent before the commencement of the study and voluntarily participated.

## 3 Results

### 3.1 Participant Characteristics

A total of 384 adults with presbycusis were included in the analysis. Demographic characteristics are summarized in Table 1. Participants had a mean age of 54.95 ± 12.47 years. Gender distribution included 180 males (46.9%) and 204 females (53.1%). Illness duration ranged from 2 to 17 years, with a mean of 6.59 ± 3.65 years. Most participants reported underlying conditions such as cardiovascular disease (64.32%) and hypertension (57.29%). Hearing aid use was reported by 302 individuals (78.64%). Additional demographic distributions—including age groups, economic status, and hospital site—are presented in Table 1.

**Table 1:** Participant demographic characteristics.

Demographic characteristics	Number		Percent
Age	Mean (SD)	54.95 (12.47)	—
Sex	Male	180	46.9
	Female	204	53.1
History of illness	Min duration	2 years	—
	Max duration	17 years	—
	Mean (SD)	6.59 (3.65)	—
Underlying disease	Diabetes 1	63	16.40
	Diabetes 2	120	31.25
	Cardiovascular disease	247	64.32
	Hypertension	220	57.29
Assistive technology	Hearing aids	302	78.64
	None	82	21.36
Smoking status	Smoker	185	48.2
Marriage status	Married	256	66.66
	Single	28	7.29
	Widowed (living alone)	100	26.04
Educational level	Non educated	54	14.06
	Elementary	105	27.3
	Secondary	106	27.6
	Diploma	41	10.6
	University degree	78	20.3
Hospital	Imam Hossein	82	21.3
	Amir Aalam	151	39.3
	Imam Khomeini	113	29.4
	Loghman	38	9.9

### 3.2 Descriptive Statistics of Main Variables

Summary statistics for personality traits, treatment adherence, and coping styles are shown in Table 2. Mean scores were 2.962 ± 0.971 for personality traits, 0.484 ± 0.363 for treatment adherence, and 2.966 ± 0.980 for coping styles.

**Table 2:** Summary statistics for main study variables.

Summary statistic	Personality traits	Adherence to treatment	Coping styles
Samples	384		
Mean ± SD	2.962 ± 0.971	0.484 ± 0.363	2.966 ± 0.980
Median	3.00	0.50	2.925
Mode	2.48	0.00	3.40
Variance	0.943	0.132	0.961
Range	3.41	1.00	3.51

### 3.3 Correlation Analysis

Spearman's correlation coefficient indicated a significant positive relationship between personality traits and treatment adherence (*r* = 0.609, *p* < 0.05), suggesting that higher personality trait scores were associated with better adherence behaviors.

### 3.4 PLS‐SEM Structural Model Results

**Figure 1:**
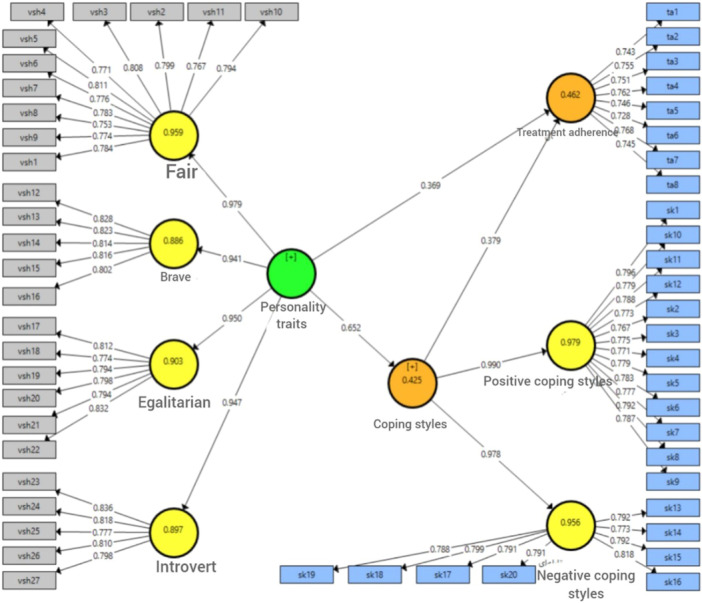
Research model of the relationship between personality traits and treatment adherence with the mediating role of coping styles in adults with presbycusis based on standard coefficients based on Partial Least Squares (PLS).

Figure 1 presents the standardized path coefficients for the hypothesized model. Personality traits demonstrated a significant positive effect on coping styles (*β* = 0.652, *p* < 0.05). Coping styles, in turn, significantly predicted treatment adherence (*β* = 0.379, *p* < 0.05). The indirect effect of personality traits on adherence through coping styles was *β* = 0.247, with a *t*‐statistic of 6.878, confirming a significant mediating pathway.

### 3.5 Model Evaluation

Model reliability, validity, and predictive accuracy indices are summarized in Table 3. All outer loadings exceeded the recommended threshold of 0.70. Composite reliability values ranged from 0.78 to 0.91, indicating satisfactory internal consistency. AVE values (0.52–0.68) supported convergent validity, and VIF values (1.22–2.41) indicated no problematic collinearity.

**Table 3:** PLS‐SEM model fit and evaluation indices.

PLS‐SEM index	Cut‐off/criterion	Obtained value	Status
Outer loadings	≥ 0.70	All ≥ 0.70	Acceptable
CR	≥ 0.70	0.78–0.91	Acceptable
AVE	≥ 0.50	0.52–0.68	Acceptable
VIF	< 5	1.22–2.41	No multicollinearity
*R* ^2^ (Coping Styles)	≥ 0.25 (weak), ≥ 0.50 (moderate)	0.42	Moderate
*R* ^2^ (Treatment Adherence)	≥ 0.25 (weak), ≥ 0.50 (moderate)	0.36	Moderate
*Q* ^2^ (Predictive relevance)	> 0	0.21–0.29	Acceptable
SRMR	< 0.08	0.046	Good fit

Predictive accuracy was moderate, with *R* ^2^ = 0.42 for coping styles and *R* ^2^ = 0.36 for treatment adherence. *Q* ^2^ values (0.21–0.29) confirmed predictive relevance. The SRMR value of 0.046 indicated good model fit within the PLS‐SEM framework.

## 4 Discussion

Presbycusis is a chronic, progressive condition that affects communication, psychosocial functioning, and cognitive health. Consistent with current rehabilitation guidelines, adults with age‐related hearing loss benefit from integrated auditory and cognitive interventions, regardless of the time elapsed since hearing aid or cochlear implant fitting [34]. Because these interventions require sustained engagement, understanding the psychological factors that shape adherence is essential.

This study demonstrated that personality traits are significantly associated with treatment adherence in adults with presbycusis, both directly and indirectly through coping styles. The findings support theoretical models suggesting that personality characteristics influence how individuals respond to chronic health demands, and that coping strategies serve as a behavioral mechanism linking personality to adherence. By integrating personality, coping, and adherence within a single structural model, this study provides empirical evidence for a pathway that has been theorized but not previously examined in presbycusis populations.

Consistent with prior research, traits such as conscientiousness, extraversion, and agreeableness appear to facilitate adherence by promoting organized behavior, proactive engagement, and social support. In contrast, individuals with higher neuroticism may experience stress, worry, or emotional reactivity that interferes with consistent treatment behaviors [15, 35]. These patterns align with findings from chronic disease populations, reinforcing the relevance of personality‐based differences in hearing‐related rehabilitation [36, 37, 38, 39].

Coping styles emerged as a significant mediator in the relationship between personality traits and adherence [22, 23]. Problem‐focused coping—characterized by active problem‐solving, seeking support, and positive adjustment—is typically associated with adaptive health behaviors. Emotion‐focused or avoidant coping, including withdrawal or distraction, may undermine adherence [24]. Our results indicate that personality traits shape the selection and use of coping strategies, which in turn influence adherence. For example, conscientious and extraverted individuals are more likely to adopt problem‐focused coping, whereas those with higher neuroticism may rely on emotion‐focused strategies [40, 41]. This mediating pathway highlights the importance of addressing coping tendencies in hearing rehabilitation programs.

These findings align with established behavioral and psychological frameworks, indicating that personality influences coping, and coping influences health behaviors [42]. They also correspond with studies linking personality traits to adherence in chronic conditions and with emerging evidence emphasizing the role of psychological factors in long‐term engagement with hearing rehabilitation [43, 44]. Recent research on presbycusis underscores the importance of stress vulnerability, coping mechanisms, and cognitive‐emotional factors in shaping treatment outcomes, further supporting the relevance of our model [6, 45, 46].

### 4.1 Implications

The results suggest that incorporating personality‐informed and coping‐focused strategies into hearing rehabilitation may enhance adherence. Clinicians may benefit from assessing personality traits and coping styles to tailor interventions, strengthen problem‐focused coping, and reduce reliance on avoidant strategies. Such approaches may improve long‐term engagement and overall quality of life for adults with presbycusis.

### 4.2 Strengths and Limitations

A key strength of this study is the use of PLS‐SEM to examine direct and indirect pathways simultaneously, providing a comprehensive understanding of psychological influences on adherence. The sample size was adequate, and model fit indices confirmed reliability and validity. However, the cross‐sectional design limits causal inference, and self‐report measures may introduce response bias. Future longitudinal studies could clarify temporal relationships and evaluate intervention strategies targeting coping styles.

## 5 Conclusion

This study demonstrates that personality traits play a meaningful role in treatment adherence among adults with presbycusis, both directly and through their influence on coping styles. Individuals who rely on positive, problem‐focused coping strategies show stronger adherence to long‐term hearing‐related interventions, whereas negative or avoidant coping may hinder consistent engagement. These findings highlight the importance of integrating psychological assessment and coping‐focused support into hearing rehabilitation programs. By addressing personality‐related tendencies and strengthening adaptive coping, clinicians may enhance adherence and improve long‐term functional and psychosocial outcomes for individuals with presbycusis.

## Author Contributions

**Zahra Davari:** writing – review and editing, writing – original draft, visualization, validation, methodology, conceptualization. **Sara Zamiran:** writing – review and editing, visualization, validation, methodology, writing – original draft. **Leila Ganji:** investigation, project administration, formal analysis. **Malek Amini:** methodology, software, formal analysis, project administration, supervision, conceptualization. **Shirin Keyvan Nasab:** data curation, project administration, formal analysis. **Marzieh Pashmdarfard:** conceptualization, writing – review and editing, supervision, formal analysis, software, validation, investigation, methodology.

## Funding

The authors have nothing to report.

## Conflicts of Interest

The authors declare no conflicts of interest.

## Transparency Statement

The Corresponding author (Marzieh Pashmdarfard) affirms that this manuscript is an honest, accurate, and transparent account of the study being reported; that no important aspects of the study have been omitted; and that any discrepancies from the study as planned (and, if relevant, registered) have been explained. All authors have read and approved the final version of the manuscript. Marzieh Pashmdarfard had full access to all of the data in this study and takes complete responsibility for the integrity of the data and the accuracy of the data analysis.

## Data Availability

The data that support the findings of this study are available from the corresponding author upon reasonable request.
